# Endometrial carcinoma: use of tracer kinetic modeling of dynamic contrast-enhanced MRI for preoperative risk assessment

**DOI:** 10.1186/s40644-022-00452-8

**Published:** 2022-03-09

**Authors:** Zhijun Ye, Gang Ning, Xuesheng Li, Tong San Koh, Huizhu Chen, Wanjing Bai, Haibo Qu

**Affiliations:** 1 Department of Radiology, Key Laboratory of Birth Defects and Related Diseases of Women and Children (Sichuan University), Ministry of Education, West China Second University Hospital, Sichuan University, No.20, Section 3, Renmin South Road, Chengdu, 610041 Sichuan China; 2 Department of Oncologic Imaging, National Cancer Center, Singapore, 169610 Singapore

**Keywords:** Endometrial neoplasms, Dynamic contrast-enhanced MRI, Risk assessment, Biomarkers, Tumor microenvironment

## Abstract

**Background:**

To compare two tracer kinetic models in predicting of preoperative risk types in endometrial carcinoma (EC) using DCE-MRI.

**Methods:**

A prospective study of patients with EC was conducted with institutional ethics approval and written informed consent. DCE-MRI data was analyzed using the extended Tofts (ET) and the distributed parameter (DP) models. DCE parameters blood flow (F), mean transit time, blood volume (Vp), extravascular extracellular volume (Ve), permeability surface area product (PS), extraction fraction, transfer constant (Ktrans), and efflux rate (Kep) between high- and low-risk EC were compared using the Mann–Whitney test. Bland–Altman analysis was utilized to compare parameter consistency and Spearman test to assess parameter correlation. Diagnostic performance of DCE parameters was analyzed by receiver-operating characteristic curve and compared with traditional MRI assessment.

**Results:**

Fifty-one patients comprised the study group. Patients with high-risk EC exhibited significantly lower Ktrans, Kep, F, Vp and PS (*P* < 0.001). ET-derived Ktrans and DP-derived F attained AUC of 0.92 and 0.91, respectively. Bland–Altman analysis showed that the consistency of Ve or Vp between the two models was low (*P* < 0.001) while Spearman test showed a strong correlation (r = 0.719, 0.871). Both Ktrans and F showed higher accuracy in predicting EC risk types than traditional MRI assessment.

**Conclusions:**

Kinetic parameters derived from DCE-MRI revealed a more hypovascular microenvironment for high risk EC than to low- risk ones, providing potential imaging biomarkers in preoperative risk assessment that might improve individualized surgical planning and management of EC.

## Background

Endometrial carcinoma (EC) is the second most common pelvic gynecologic malignancy in China [1] and the most common pelvic gynecological malignancy in North America and Europe [2, 3]. In recent years, there has been a steady rise in the incidence of EC in developing countries probably due to gradual adoption of a Western lifestyle and increased life expectancy [4]. The recommended standard surgical procedure includes extrafascial total hysterectomy with bilateral salpingo-oophorectomy. Studies have shown that pelvic and para-aortic lymphadenectomy is considered to be of therapeutic value in patients at high risk of recurrence, while being of limited value in low-risk patients [5–7]. Histologic grade, deep myometrial invasion (DMI), cervical stroma invasion (CSI), lymphovascular space invasion (LVSI), and lymph node metastases (LNM) are important prognostic factors and are used for risk stratification [8–11]. However, these prognostic factors can only be accurately assessed in surgical specimens, and simultaneous lymphadenectomy and hysterectomy may overtreat low-risk patients. Therefore, a diagnostic tool based on understanding the tumor microenvironment to aid in preoperative risk assessment is desirable in a clinical setting.

Dynamic contrast-enhanced (DCE) magnetic resonance imaging (MRI) is a potential tool for early diagnosis and identification of endometrial cancer, and predicting tumour treatment response using semi-quantitative and quantitative pharmacokinetic methods [12–16]. Although modeling with parametric techniques is complex and computationally demanding, this approach has been demonstrated to be preferable to the model-free approach [17]. There are two main categories of DCE-MRI kinetic models, namely compartmental and spatially distributed models. The compartmental model describes the exchange of the contrast agent (CA) within the compartments and assumes that it is well-mixed, the ouput CA flux in any compartment is proportional to the concentration. The spatially distributed model, on the other hand, describes a more realistic flow model that assumes intravascular and extracellular extravascular spaces and allows for the exchange of CA only in the vicinity of the capillary bed. Unlike the compartmental model, the spatially distributed model takes into account both spatial and temporal variations. In EC, some DCE-MRI kinetic models have been demonstrated to be effective in predicting known prognostic factors (staging, histologic grade, subtype, etc.) and outcomes [18–23], with the the extended Tofts (ET) [24–29] model being widely used as a compartment model. The distributed parameters (DP) [30–32] model, despite being the first type of the spatially distributed model can theoretically provide a more realistic response to the tumour microenvironment, is not yet widely used due to its higher complexity. Both of the two DCE models consist of two compartments describing the intravascular and extravascular spaces of the tissue. However, there are fundamental differences in the two models: (i) the ET model assumes homogeneous tracer concentration in both compartments, whereas the DP model accounts for concentration gradients in the compartments. (ii) the ET model describes the transport processes of blood flow and vascular-interstital exchange by a single transfer constant, Ktrans; while the DP model separately accounts for these transport processes with individual parameters for blood flow, F and permeability-surface area product, PS. This study was designed with high temporal resolution and sufficient acquisition time to be able to meet the assumptions of both models. To the best of our knowledge, there are no studies on DCE-MRI models for the prediction of risk types of EC. Moreover, there is a lack of studies to guide the use of different tracer kinetic models in EC. The aim of this study is to explore the value of these two models in EC risk type prediction compared with traditional MRI.

## Materials and methods

### Patients

This study was approved by the ethics committee of West China Second University Hospital, Sichuan University (NO. 200), and informed consent was obtained from each patient. Women with biopsy-proven EC were recruited prospectively. All patients were initially diagnosed with EC by hysteroscopic biopsy or dilatation and curettage, and patient demographic characteristics, treatment modalities, postoperative histological subtype and classification according to the revised International Federation of Gynecology and Obstetrics (FIGO) staging were recorded.

### MRI protocol

MRI scanning was performed on a 1.5 T MR (Achieva Nova Dual, Philips Healthcare Best, Netherlands) using a 16-channel body coil. The imaging protocol included both morphological (standard diagnostic MRI based on the Guidelines of the European Society of Urogenital Imaging [33]) and functional imaging (DCE-MRI).

The morphological pelvic MR imaging consisted of sagittal, oblique axial and coronal axial T2-weighted images, oblique axial T1-weighted images before and after an intravenous bolus injection of 0.2 mL/kg of gadobutrol (Magnevist, Bayer Healthcare, Leverkusen, Germany) administered at 2 mL/s injection speed.

The DCE-MRI measurement was performed using the three-dimensional T1-weighted High-Resolution Isotropic Volume Excitation (THRIVE) sequence in the oblique axial plane. Acquisition parameters were as follows: echo time 2.1 ms, repetition time 4.4 ms, flip angle 18°, matrix 285 × 285, field of view 375 × 375 mm^2^, 9 slices, slice thickness 5 mm, number of averages 1, average temporal resolution 2.5 s, 10 precontrast scans for each flip angle 6°, 12°, and 18° [34], and 115 postcontrast scans with flip angle 18°.

### Histological diagnosis and immunohistochemistry

*Assessment of tumor risk type*—Surgical specimens were examined by a gynecologic oncological pathologist (*BLINDED*, with 38 years of experience). According to the FIGO Cancer Report 2018, patients with grade 1–2 endometrioid adenocarcinoma without DMI, CSI, LVSI, and LNM were classified as low-risk disease, whereas those with endometrioid grade 3, nonendometrioid, DMI, CSI, LVS, or LNM were classified as high-risk disease.

*Assessment of microvessel density*—Immunohistochemical staining was performed with factor CD105 enabling calculation of microvessel density (MVD). Sections were first examined at low magnifications (× 100) to identify the most vascular areas of the tumor. Subsequently, MVD was counted in each field (× 400; field size 0.145mm^2^), and the median of 10 fields was estimated for each patient.

### Image analysis

*Assessment of DCE-MRI parameters*—Anonymized DCE-MR images were exported and analyzed offline using a commercially-available software (Mltalytics, FITPU Healthcare, Singapore). The following parameters were obtained with the ET model: transfer constant (Ktrans; min^− 1^), efflux rate constant (Kep; min^− 1^), extravascular extracellular volume (Ve; mL/100 mL), and blood volume (Vp; mL/100 mL). The following parameters were obtained with the DP model: blood flow (F; mL/min/100 mL), extraction fraction (E; %), permeability surface area product (PS; mL/min/100 mL), Vp, Ve, and mean transit time (MTT; s).

To derive the arterial input function (AIF) and delineate the tumor, region of interest (ROI) analysis was independently performed by two radiologists (*BLINDED* and *BLINDED*, with 10 and 5 years of experience in pelvic MRI, respectively). The readers were blinded to tumor stage, histologic diagnosis, and patient outcome. The tumor ROI was manually selected layer by layer on the level of the DCE-MR images containing tumor tissue, avoiding necrotic or hemorrhagic areas. Morphological images were cross-referenced to verify anatomical structures and tumor location. Tissue concentration–time curve corresponding to each voxel within the tumor ROI was fitted separately using the ET and DP models. Because the AIF varies with cardiac function, individual subject AIF was used. Although there are smaller arteries closer to the cervix cancer, these smaller arteries might not be visible in the DCE scans. Also, if the artery could not occupy a voxel entirely, partial volume effect could result in a lower AIF. The iliac artery was chosen to avoid possible partial volume effect. Median parameter values were recorded for each patient.

*Traditional MRI visual assessment*—Two radiologists (*BLINDED* and *BLINDED*, with 20 and 8 years of experience in pelvic MRI, respectively), blinded to clinical data and histological results, evaluated the MR images independently using an open source software (Horos v3.3.6, Horosproject.org). One of the radiologists (*BLINDED*) was involved in the drawing of ROIs on DCE-MRI. Visual assessment was performed 4 months after ROIs were drawn to avoid possible recall bias. Each reader interpreted the presence of DMI, CSI and LNM with a binary score using T2-weighted and DCE images independently. Differing results from traditional MRI were discussed and the consensus opinion was recorded. On T2-weighted images, DMI was defined as tumour involvement of moderate signal myometrium to 50% or more of the myometrial thickness [35], CSI was defined as hypointense cervical stroma disrupted by an intermediately intense or hyperintense tumor [36]. On the DCE MR images, DMI was defined as the tumor involves 50% or more of the enhanced myometrial thickness, CSI was defined as interruption of the enhancement of the normal cervical epithelium [37]. LNM is largely based on size criteria, with a short axis diameter of greater than 8 mm in pelvic nodes and 10 mm in para-aortic nodes is taken to indicate tumour involvement [38]. Other morphological features including round shape, spiculated margins, signal intensity similar to the primary tumor, abnormal enhancement on DCE images, or the presence of necrosis can suggest small lymph node involvement [39].

### Statistical analysis

Statistical analyses were performed using Prism 8.4.0 (GraphPad Software, La Jolla, CA, USA). The reported statistical significance levels were two sided, and *P* < 0.05 was considered significantly different. Reader agreement regarding the ROIs was analyzed using the ﻿intraclass correlation coefficient *(ICC* < 0.40 indicating poor agreement; 0.40 ≤ *ICC* ≤ 0.75 good agreement; *ICC* > 0.75 excellent agreement). For all derived imaging parameters, median values with 95% confidence interval of the median were calculated. Bland–Altman plots were used to evaluate the consistency of similar parameters from different models, and parameter difference was examined using the Wilcoxon signed-rank test. Spearman’s correlation test was used to assess the correlation between the DCE parameters derived from the two models and the correlation between the DCE parameters and MVD. A Mann-Whitney U test was used to evaluate the differences between low- and high-risk type for each imaging parameter. The method of False Discovery Rate (FDR) correction was utilized to correct *p*-values in multiple tests. The receiver-operating characteristic (ROC) curve was employed to evaluate the diagnostic performance of each parameter of the two models and MVD for predicting risk types, which was quantified using the area under the ROC curve (AUC). Optimal cut-off values were chosen using the Youden index on the estimated curves. The McNemar test was performed to compare the diagnostic performance of the DCE-MRI models to that of traditional MRI assessment for risk type.

## Results

**Fig. 1:**
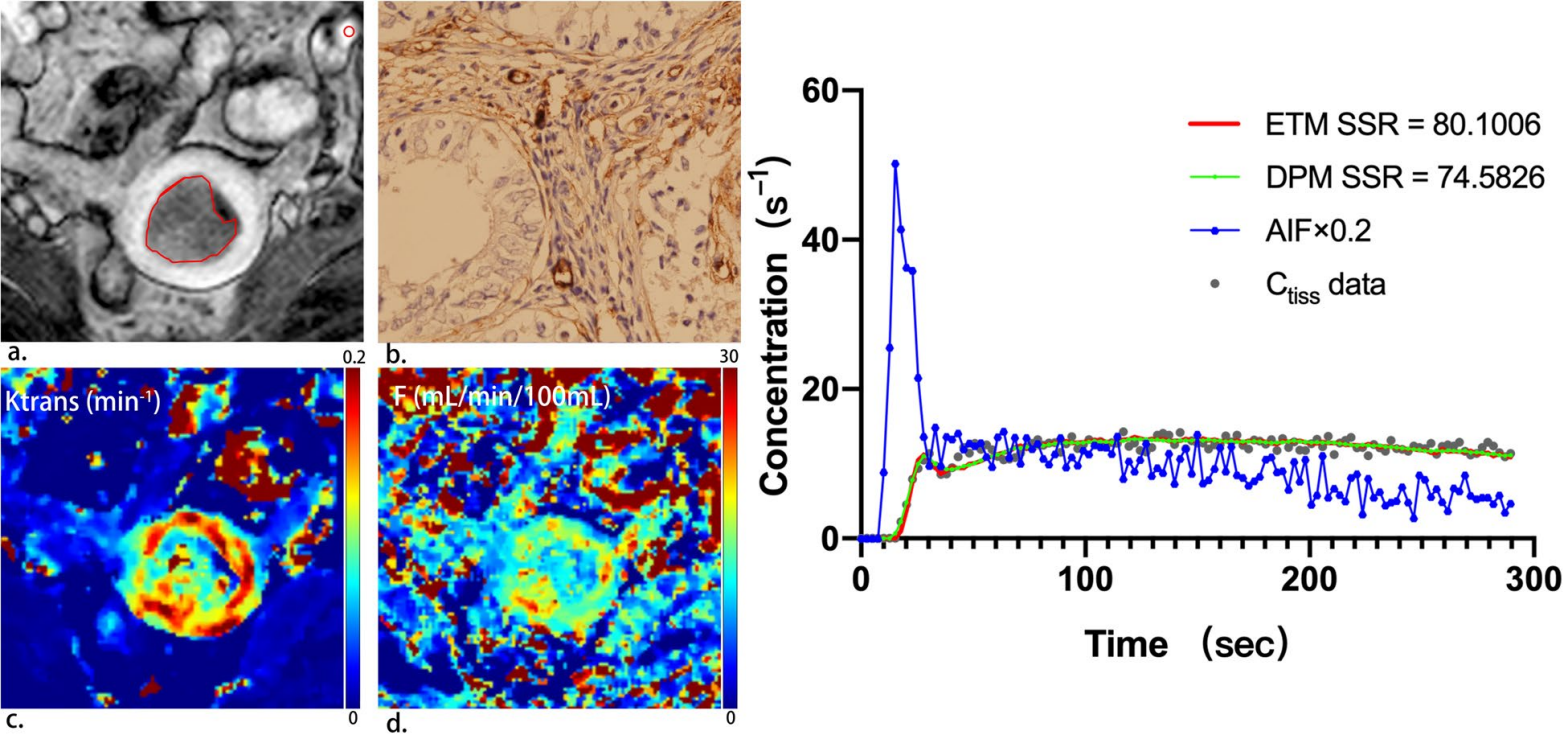
A 53-year-old patient with low-risk endometrial carcinoma. (**a**) On oblique axial DCE-MRI, tumor is delineated by red line and AIF is delineated by red circle. (**b**) Immunohistochemical staining with CD105 (magnification 400) showed 14 microvessels per mm^2^ indicating low microvessel density in the tumour. (**c**) Parametric map of transfer constant (Ktrans) for the extended Tofts (ET) model. The mean value of Ktrans for the tumor was 0.12 min^-1^, indicating relatively high permeability. (**d**) Parametric map of blood flow (F) for the distributed parameter (DP) model. The mean value of F for the tumor was 13.74 mL/min/100 mL, indicating relatively high blood flow. (**e**) Example of fitting a voxel tissue concentration (Ctiss)–time curve with the extended Tofts (ET) model and the distributed parameter (DP) model (right panel). Smaller sum-of-squared residue (SSR) is indicative of better fit. The ET and the DP model yield similar SSR values

**Fig. 2:**
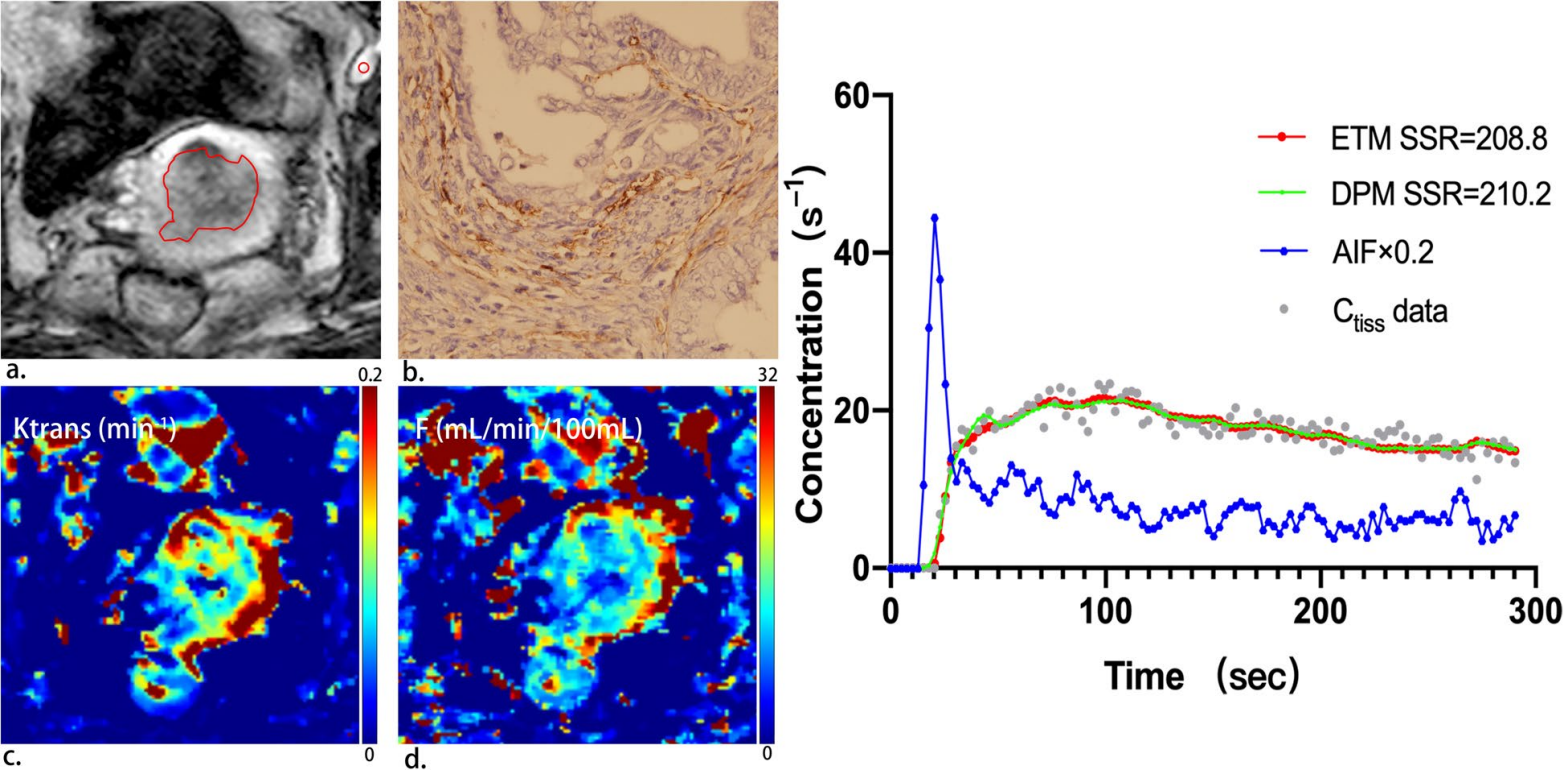
A 65-year-old patient with high-risk endometrial carcinoma. (**a**) On oblique axial DCE-MRI, tumor is delineated by red line and AIF is delineated by red circle. (**b**) Immunohistochemical staining with CD105 (magnification 400) showed 41 microvessels per mm^2^ indicating high microvessel density in the tumour. (**c**) Parametric map of transfer constant (Ktrans) for the extended Tofts (ET) model. The mean value of Ktrans for the tumor was 0.08 min^-1^, indicating relatively low permeability. (**d**) Parametric map of blood flow (F) for the distributed parameter (DP) model. The mean value of F for the tumor was 10.21 mL/min/100 mL, indicating relatively low blood flow. (**e**) Example of fitting a voxel tissue concentration (Ctiss)–time curve with the extended Tofts (ET) model and the distributed parameter (DP) model (right panel). Smaller sum-of-squared residue (SSR) is indicative of better fit. The ET and the DP model yield similar SSR values

A total of 112 patients were prospectively recruited; 61 patients were excluded [lesions were too small for delineation or were not visualized on MRI (*n* = 33), refusal of inclusion (*n* = 9), change of therapy (*n* = 7), final pathology finding confirmed not to be EC (*n* = 6), noisy images or hence failure to sample AIF (*n* = 6)]. The remaining 51 patients (median age, 55 years; range, 20–75 years) comprised the study group. All patients were diagnosed with primary EC and were surgically staged according to the FIGO staging system (2018). Table 1 summarizes these patient characteristics. Figure 1 provides an example of a low risk EC patient with representative parameteric maps generated using the ET and DP models. An example of a high risk EC patient is shown in Fig. 2.

**Table 1:** Clinicopathological patient characteristics

Clinicopathological patient characteristics	
Age; mean (range) years	55 (20–75)
Postmenopausal, n	33
**Histological subtype, n**	
Endometrioid	31
Grade 1	18
Grade 2	9
Grade 3	4
Nonendometrioid	20
**DMI, n**	
Yes	11
No	40
**CSI, n**	
Yes	4
No	47
**LVSI, n**	
Yes	8
No	43
**LNM, n**	
Yes	5
No	46

*DMI* deep myometrial invasion, *CSI* cervical stroma invasion, *LVSI* lymphovascular space invasion, *LNM* lymph node metastases

### Comparison of DCE kinetic parameters and MVD

**Fig. 3:**
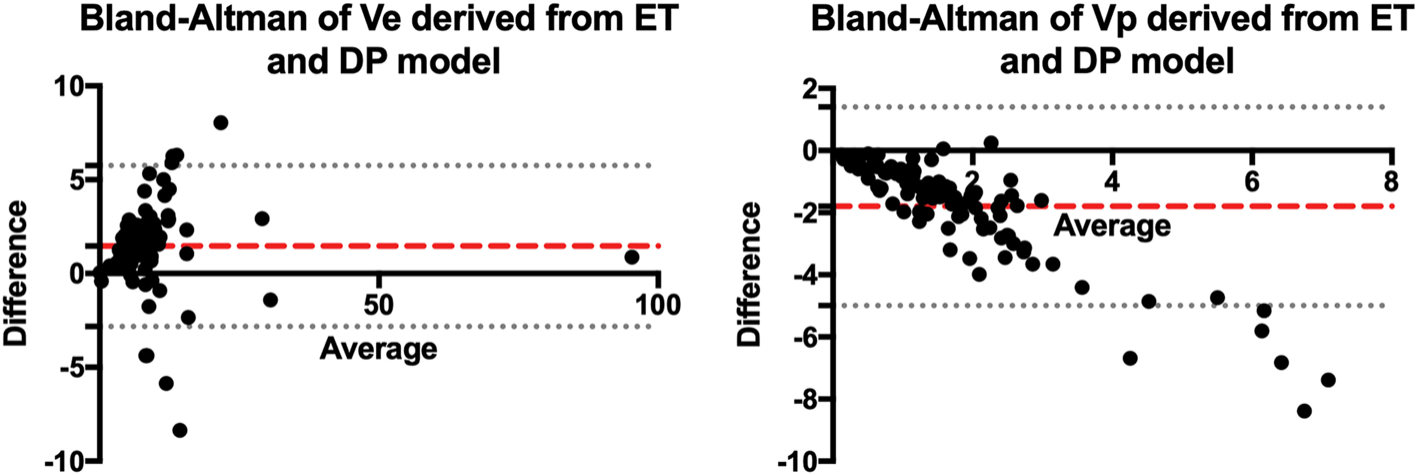
Bland–Altman analysis of Ve (left panel) and Vp (right panel)derived from the ET and the DP model. Difference refers to ET minus DP parameters. ET = extended Tofts, DP = distributed parameter, Ve = extravascular extracellular volume, Vp = blood volume

**Fig. 4:**
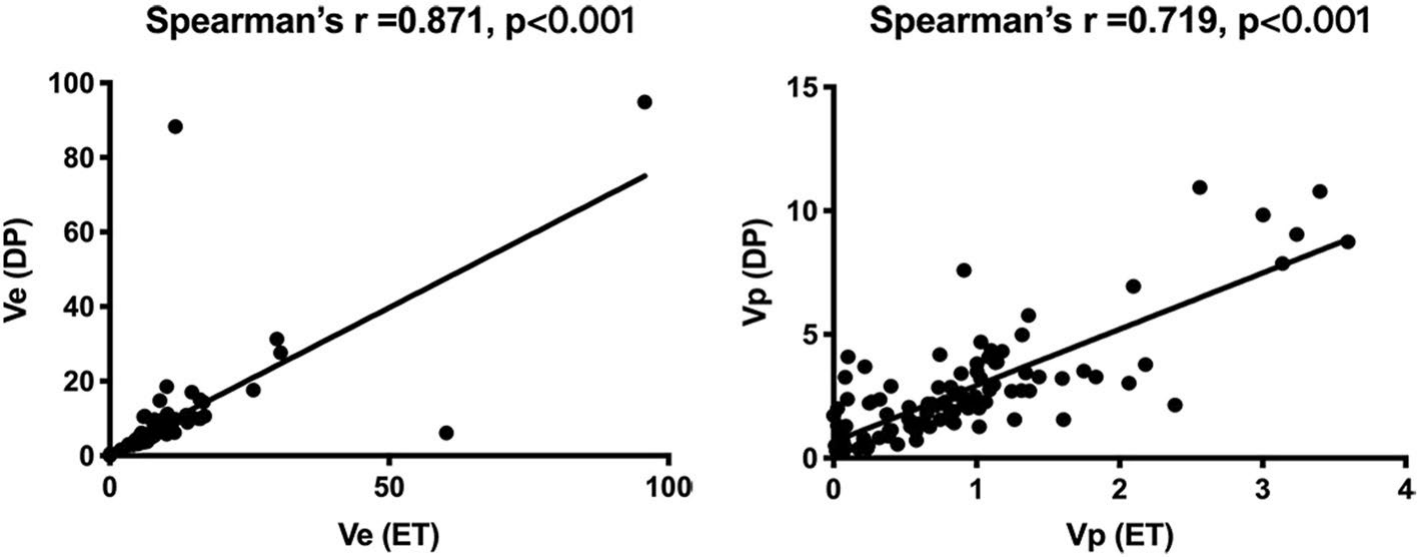
Spearman correlation test of Ve(left panel) and Vp (right panel) derived from the ET and the DP model. ET = extended Tofts, DP = distributed parameter, Ve = extravascular extracellular volume, Vp = blood volume

The median ROI size assessed by structural MRI was 40 voxels (mean, 78 voxels; range, 6–800 voxels). ROI agreement rate between readers ranged from good (*ICC* = 0.715) to excellent (*ICC* = 0.952). The median values of DCE kinetic parameters did not follow a normal distribution, and Table 2 showed the range and median parameter values in low- and high-risk type of EC. Both models estimated parameters Ve and Vp. Bland–Altman analysis (ET-DP parameter vs average of ET and DP parameter) showed that the consistency of either Ve or Vp between ET and DP estimates was very low (*P* < 0.001 for Ve, *P* < 0.001 for Vp). A negative trend was evident in the Bland–Altman plot of Vp (Fig. 3), indicating a larger deviation in DP-derived Vp than in ET-derived Vp. Spearman correlation analyses between ET and DP parameters yielded strong and significant correlations between ET-derived Ktrans with DP-derived F (*r* = 0.782, *P* < 0.001), PS (*r* = 0.755, *P* < 0.001), and Vp (*r* = 0.658, *P* < 0.001). Although the values of Vp and Ve as estimated by ET and DP differed significantly (Fig. 3), they were strongly correlated (*r* = 0.719, *P* < 0.001; *r* = 0.871, *P* < 0.001) (Fig. 4). The other parameters from the two models were weakly correlated. The DCE parameters and MVD are weakly correlated. The median MVD was 55 (mean, 52; range, 10–131) microvessels per mm^2^ and 90 (mean, 90;range, 24–193) microvessels per mm^2^, respectively, for low- and high-risk type of EC (*P* < 0.001).

**Table 2:** Parameter values and *P* values derived with the ET and DP models

	Low-risk type Median (95% CI)	High-risk type Median (95% CI)	***P*** value	Corrected ***P*** value
**ET model**				
Ktrans	0.10 (0.09–0.12)	0.05 (0.04–0.05)	< 0.001	< 0.001^*^
Vp	1.02 (0.82–1.14)	0.58 (0.38–0.74)	< 0.001	< 0.001*
Ve	8.12 (6.50–10.23)	7.86 (6.95–8.96)	0.39	0.39
Kep	1.20 (0.94–1.44)	0.57 (0.51–0.68)	< 0.001	< 0.001*
**DP model**				
F	13.01 (12.26–14.79)	8.12 (6.86–8.74)	< 0.001	< 0.001*
MTT	15.15 (12.12–17.33)	12.38 (11.03–14.19)	0.08	0.13
Vp	3.03 (2.37–3.69)	1.46 (1.22–2.02)	< 0.001	< 0.001*
Ve	6.17 (4.88–8.06)	6.31 (5.74–8.09)	0.61	0.61
PS	8.32 (6.75–10.74)	4.01 (3.16–5.14)	< 0.001	< 0.001*
E	43.63 (37.02–51.29)	38.72 (34.42–47.26)	0.52	0.63

^*^ Significant *P* values

*ET* extended Tofts, *DP* distributed parameter, *Ktrans* transfer constant, *Kep* efflux rate constant, *Vp* blood volume, *Ve* extravascular extracellular volume, *F* blood flow, *MTT* mean transit time, *PS* permeability surface area product, *E* extraction fraction. Ktrans and Kep are in units of min^−1^, Vp and Ve are in units of mL/100 mL, F and PS are in units of mL/min/100 mL, MTT is in unit of seconds, E is in unit of %

### Diagnostic performance in predicting risk types

**Fig. 5:**
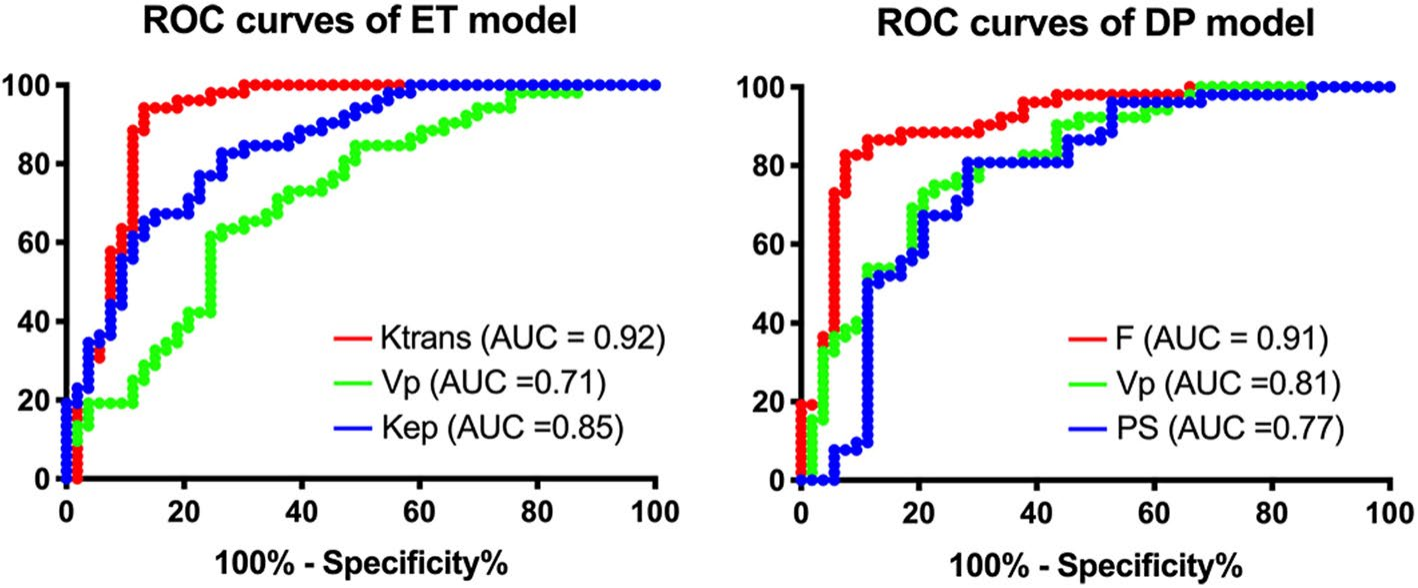
The ROC of top three performed parameters in the ET (left panel) and the DP (right panel) model. ROC = receiver-operating characteristic curve, ET = extended Tofts, DP = distributed parameter, Ktrans = transfer constant, Vp = blood volume, Kep = efflux rate constant, F = blood flow, PS = permeability surface area product

Significantly lower values of Ktrans (*P* < 0.001), Vp (*P* < 0.001) and Kep (*P* < 0.001) in ET model, and significantly lower values of F (*P* < 0.001), Vp (*P* < 0.001) and PS (*P* < 0.001) in DP model were observed in high-risk type than in low risk type of EC (Table 2). Figure 5 presented the ROC analysis of these parameters for ET and DP. Table 3 summarises the AUC, sensitivity, specificity and accuracy of ET, DP and MVD. In pairwise comparisons of the DCE models and the traditional MRI, DCE-MRI parameters Ktrans and F showed greater accuracies and sensitivities than tradtional MRI (*P* < 0.05), and the differences in specificities were not statistically significant (*P* > 0.1) (Table 4).

**Table 3:** Comparison of diagnostic parameters for predicting risk types

Variable	ET model			DP model			MVD
	Ktrans	Vp	Kep	F	Vp	PS	
**AUC**	0.92 (0.86–0.98)	0.71 (0.62–0.81)	0.85 (0.77–0.92)	0.91 (0.85–0.97)	0.81 (0.73–0.90)	0.77 (0.68–0.87)	0.79 (0.70–0.87)
**Accuracy** **(%)**	90	69	78	88	76	76	76
**Sensitivity (%)**	94	63	74	87	77	72	65
**Specificity (%)**	87	74	83	89	75	81	82

Data in parentheses are 95% confidence interval range

*ET* extended Tofts, *DP* distributed parameter, *Ktrans* transfer constant, *Kep* efflux rate constant, *Vp* blood volume, *F* blood flow, *PS* permeability surface area product, *MVD* microvessel density. Ktrans and Kep are in units of min^−1^, Vp is in units of mL/100 mL, F and PS are in units of mL/min/100 mL, MVD are in units of microvessels per mm^2^. AUC = area under the receiver operating characteristic curve

**Table 4:** Diagnostic performace of DCE models and traditional MRI for assessment of endometrial cancer risk types

Variable	Ktrans (ET)	F (DP)	DMI	CSI	LNM
**Accuracy (%)**	90	88	63 (0.011, 0.004)	59 (0.003, 0.001)	63 (0.007, 0.004)
**Sensitivity (%)**	94	87	58 (0.039, 0.039)	19 (0.001, 0,001)	31 (0.001, 0.001)
**Specificity (%)**	87	89	68 (0.227, 0.109)	100 (0.250, 0.500)	96 (0.625, 1.000)

The first P value is compared with Ktrans, the second *P* value is compared with F. *ET* extended Tofts, *DP* distributed parameter. Ktrans = transfer constant, in units of min^− 1^, F = blood flow, in units of mL/min/100 mL, *DMI* deep myometrial invasion, *CSI* cervical stroma invasion, *LNM* lymph node metastases

## Discussion

Our study found that tracer kinetic analysis of DCE-MRI data can differentiate the tumor microenvironment of different risk types of EC. High-risk ECs were found to exhibit lower F, Ktrans, PS, and Vp than low-risk ECs. Although the parameter values estimated by ET and DP models were significantly different, the two models yielded similar diagnostic performance in distinguishing low-risk from high-risk ECs. The DCE-MRI model is not yet widely used and its application in EC is mainly focused on individual risk factors [24, 40]. There have also been studies using DCE-MRI models to preliminary explore the microenvironment and prognosis of EC, but they are of limited help in preoperative risk assessment and lack comparison with traditional MRI [26, 27]. EC risk type takes in our study into account a variety of recognized preoperative risk stratification factors: pathological type of tumor, DMI, CSI, LVSI, and LNM [8–10]. These factors have been widely used to stratify patients based on recurrence risk for recommending pelvic and para-aortic lymphadenectomy in high-risk patients [5–7]. For predicting risk type, the diagnostic accuracy and sensitivity of the DCE-MRI tracer kinetic model were superior to experienced radiologists in gynecologic oncology, with comparable diagnostic specificity. Our qualitative diagnostic performance for traditional MRI was slightly lower than that in previous reports [41, 42]. This can be partially explained by the fact that we excluded 33 (29.5%) of 112 patients whose tumors were too small to accurately determine tumor outlines. Most of these small tumors were confined to the endometrium or exhibited minimal invasiveness, and inclusion may improve the diagnostic performance of traditional MR imaging. The human eye is unable to detect the presence of a particular pathological type and LVSI on MR images. Although hysteroscopic with biopsy or curettage is recommended for preoperative determination of the pathological type, it is rarely suggestive of pathological grading; and intraoperative frozen sections are commonly used to select patients suitable for extensive surgery [43], which has limited benefit for low-risk patients [44]. Sentinel-lymph node (SLN) mapping has reported encouraging results in detecting EC metastasis [45]; however, on one hand, this is an additional intraoperative operation in which the surgeon’s expertise and attention to technical details are crucial. On the other hand, staging without lymph node pathological findings may increase the risk of postoperative chemoradiotherapy in patients. For cases of failed SLN mapping, preoperative evaluation of the tumor still needs to be used to guide treatment. This study, in addition to aiding in predicting patient risk preoperatively, also provides information on tumor microcirculation, which will likely help us understand how endometrial cancer grows and also provide assistance for possible adjuvant therapy.

In recent studies of EC, analysis of DCE MRI data have been performed using semi-quantitative tissue enhancement metrics (such as maximum slope, maximum concentration and time-to-peak) [18] and the ET model [26–28, 40]. Although the ET model is fast to compute, its parameter Ktrans reflects a combination of tumor blood flow and vessel permeability, and it is unclear which of the two processes (blood flow or blood-tissue exchange) resulted in the measured Ktrans. The DP model separately accounts for blood flow F and vessel permeability PS, but it is mathematically more complex and requires longer computation time. To the best of our knowledge, application of the DP model on DCE MRI data of EC has not been explored. Our study found that the diagnostic performance of the two tracer kinetic models was very close. Both models attained good performance in terms of the characterization of EC risk type, where Ktrans and F yielded AUC values > 0.90, indicating that the simple ET model is well applicable for differentiating EC risk type. It is interesting to see that though the AUCs of Ktrans and F were nearly equal, the former seems to favour sensitivity and the latter specificity. In addition, ET-derived Ktrans showed strong correlation with DP-derived F, PS, and Vp. Nevertheless, each tracer kinetic model makes certain assumptions, resulting in measurements of different parameters or different values for the same parameter. Thus, the results of the two models should be interpreted with care.

The DP model can be expressed in two phases of the capillary–tissue system: vascular phase and interstitial reflux phase [32]. The imaging protocol in the present study consisted of an adequate temporal resolution (2.5 s) and sufficient acquisition time (4 min 50 s) for capturing these two phases. In comparison, although ET is a two-compartment model, the combined effects of blood flow and vascular-tissue exchange were described by Ktrans. In this study, DP model revealed reduced F and reduced permeability in high-risk ECs. Previous studies [46, 47] showed that the reduced blood flow leads to a reduced oxygen supply, which is inherent in various pathogenetic mechanisms, leading to either temporary or chronic hypoxia. Therefore, the measured values of lower flow and lower permeability in high-risk types might suggest a more hypoxic condition in high-risk EC than in low-risk EC. Cells in hypoxic conditions tend to be unresponsive to chemotherapeutic reagents. Tumor hypoxia is an independent prognostic indicator of poor patient outcome [48]. Thus, separate assessment of blood flow and permeability would be of clinical importance and might result in a better understanding of tumor biology and the relationship between perfusion and hypoxia in tumor tissue, which is valuable in radiation oncology and might potentially aid in the development of novel treatment strategies in tumor management by imaging.

Haldorsen et al. applied the adiabatic tissue homogeneity (ATH) model in 55 patients with EC and showed that F and capillary transit time were correlated with preoperative risk stratification factors; in particular, low tumor F was a poor prognostic factor [26, 27]. The authors observed that reduced F was accompanied by increased capillary leakage in tumors with high microvascular proliferation [26]. However, the present study demonstrated that high-risk ECs exhibited lower F and lower capillary leakage (PS) than low-risk ECs. In addition to the use of different tracer kinetic models (ATH and DP), the difference could be attributed to the choice of AIF: a population AIF was used in the previous study [27], whereas the current study used patient-specific AIF.

MVD has been shown to be a histomorphological marker of tumor angiogenesis and is significantly associated with tumor proliferation and prognosis in EC [49]. Our study found that MVD attained the AUC value of 0.79 in distinguishing patients with low- and high-risk types. However, MVD cannot be assessed preoperatively, and tumor heterogeneity makes it prone to sampling errors in larger tumors. In addition, our findings of significantly lower Vp and significantly higher MVD in high-risk ECs are apparently contradictory to established facts of tumor angiogenesis, which typically generates numerous and leaky vessels. Zinovkin et al. [50] found a significantly low overall survival rate of patients whose tumors had > 114 vessels/mm^2^ of tissue. Reijnen et al. [49] showed that adverse outcomes in hypoxic ECs were observed in the presence of high vascular density, which is baffling in terms of the relationship between tissue hypoxia and vascular density in EC. The lack of congruence between these two factors might explain the ostensible mismatch between the DCE-measured Vp and immunohistochemically measured vasculature. In fact, some tumors do not possess features of numerous and leaky vessels. It has been observed that mature and stable vessels are essentially functional. Further, leaky vasculature is caused when the tumor reaches a size that it requires increased blood supply for its growth, i.e., mid- to late-stages [51, 52]. Increased viscosity and geometric resistance due to vascular disorganization can affect tumor blood flow. As a result, the average erythrocyte flow rate in tumor vessels may be an order of magnitude lower than in normal vessels, and the overall tumor perfusion rate is reduced compared to many normal tissues [53]. In addition, the structure of the tumor vasculature is heterogeneous and irregular, and proliferating tumor cells and/or stromal cells exert elevated interstitial pressure on the vasculature, which may lead to vascular collapse [54]. Thus, the DCE-measured vasculature might correlate to the true functionality of tumor vessels, which could become dysfunctional in EC.

This study has some limitations. First, this was a prospective analysis in a relatively small number of patients, and the findings should be further validated in a larger cohort. Second, the DCE MR images were acquired with a slice thickness of 5 mm (to increase SNR in the dynamic scans) which is thicker than the usual anatomical (diagnostic) MR scans of 1-2 mm. ROIs for small tumors (~ 5-10 mm) can only be identified on one or two DCE slices, and the corresponding results could be prone to errors related to partial volume and intratumoral heterogeneity effects. Third, instead of analyzing various risk factors separately, we combined them into two types, which could lead to an increase in confounders of outcomes. However, the inclusion of recognized major risk factors can simplify clinical procedures and provide more intuitive results for surgical planning.

## Conclusions

DCE-MRI was applied for the assessment of EC risk types, and the results revealed that high-risk ECs are characterized by low blood flow (F), low permeability (PS), low transfer constant (Ktrans), and low functional vascularity (Vp). Excellent diagnostic performance is attained by ET-derived Ktrans and DP-derived F. These encouraging results warrant further studies to standardize tracer kinetic modelling and DCE-MRI as a potential imaging tool for preoperative risk stratification in patients with ECs.

## Data Availability

The datasets used and analysed during the current study are available from the corresponding author on reasonable request.

## Abbreviations

AIF: Arterial input function
AUC: Area under curve
CSI: Cervical stroma invasion
DP: Distributed parameter
E: Extraction fraction
EC: Endometrial carcinoma
EES: Extravascular extracellular space
ET: Extended Tofts
DCE: Dynamic contrast-enhanced
DMI: Deep myometrial invasion
F: Blood flow
LNM: Lymph node metastases
LVSI: Lymphovascular space invasion
MRI: Magnetic resonance imaging
MTT: Mean transit time
MVD: Microvessel density
PS: Permeability-surface area product
ROC: Receiver-operating characteristic
ROI: Region of interest
Vp: Blood volume
Ve: Extravascular extracellular volume
